# Hierarchical and hybrid energy storage devices in data centers: Architecture, control and provisioning

**DOI:** 10.1371/journal.pone.0191450

**Published:** 2018-01-19

**Authors:** Mengshu Sun, Yuankun Xue, Paul Bogdan, Jian Tang, Yanzhi Wang, Xue Lin

**Affiliations:** 1 Department of Electrical and Computer Engineering, Northeastern University, Boston, Massachusetts, United States of America; 2 Ming Hsieh Department of Electrical Engineering, University of Southern California, Los Angeles, California, United States of America; 3 Department of Electrical Engineering and Computer Science, Syracuse University, Syracuse, New York, United States of America; Chongqing University, CHINA

## Abstract

Recently, a new approach has been introduced that leverages and over-provisions energy storage devices (ESDs) in data centers for performing power capping and facilitating capex/opex reductions, without performance overhead. To fully realize the potential benefits of the hierarchical ESD structure, we propose a comprehensive design, control, and provisioning framework including (i) designing power delivery architecture supporting hierarchical ESD structure and hybrid ESDs for some levels, as well as (ii) control and provisioning of the hierarchical ESD structure including run-time ESD charging/discharging control and design-time determination of ESD types, homogeneous/hybrid options, ESD provisioning at each level. Experiments have been conducted using real Google data center workloads based on realistic data center specifications.

## Introduction

Modern data center investments comprise one-time infrastructure costs that are amortized over the lifetime of data center (capex) and monthly recurring operating expenses (opex) [1]. The capex is directly impacted by data center’s peak power requirement, which determines the provisioned capacity of power infrastructure and is estimated at $10-20 per Watt [2]. The opex is charged by utility company based on high power tariff scheme and dynamic energy pricing policy [3], and has been steadily increasing [4]. Reducing both capex and opex of a data center has become a key enabler to ensure its economic success.

Because (i) the capex depends on the largest provisioning power and (ii) up to 40% opex is caused by the peak power tariff [5], *power capping* is widely studied for modern data centers to reduce the peak power, thereby simultaneously reducing the capex and opex. Majority of power capping techniques focus on (i) throttling computing devices [6, 7], (ii) shifting the workload peak draw temporally/spatially [8, 9], and (iii) improving non-peak/idle power efficiency of servers [10–14]. These solutions can have adverse performance consequences depending on the workload behavior. This will become a problem for any workload that has performance constraints or service-level agreements.

Recently, a new approach has been introduced that leverages and over-provisions energy storage devices (ESDs) in data centers for facilitating capex/opex reductions [2, 3, 5, 15, 16], without performance overhead. After over-provisioning, ESDs can be leveraged for power capping [2] and peak power shaving [5, 17]. Reference work [2, 5, 17] have demonstrated that the benefits from capex and opex reductions outweigh the extra costs associated with ESD over-provisioning.

ESDs in data center are commonly made of lead-acid batteries and utilized as centralized uninterruptible power supply (UPS) [18, 19]. Potential ESDs also include Li-ion batteries, supercapacitors, flywheels, and compressed air energy storage (CAES). Lead-acid and Li-ion the are the most widely adopted ESDs, due to their good reliability and high energy density [20–22]. Particularly, Li-ion batteries have significantly high energy density, high efficiency, long cycle life, and environmental friendliness, and therefore is one of the most promising technologies in electrical energy storage [19]. Supercapacitors have much higher power density due to the electrochemical double-layer structure [21, 22]. Flywheels depend on the momentum of rotating wheel/cylinder to provide temporary power [22], while air in CAES is compressed to store electrical energy and decompressed to discharge energy [3, 22]. Some key characteristics for various types of ESDs are listed in Table 1 with the data derived from [20–22], in order to scrutinize and select the most appropriate ESDs in data centers. Most applications employ a single energy storage technology, while for certain applications with more advanced requirements, it is desirable to use two or more ESDs with complementary characteristics, either by combining different batteries or by integrating a battery with a supercapacitor, flywheel, etc [19].

**Table 1:** Summary of key characteristics of ESDs.

	Cycle efficiency	Unit capital cost ($/kWh)	Self-discharge per day	Energy density (Wh/kg)	Power density (W/kg)	Cycle life
Lead-acid battery	70∼90%	100∼200	0.1∼0.3%	30∼50	75∼300	500∼800
Li-ion battery	∼90%	600∼1500	0.1∼0.3%	100∼250	250∼340	1000∼5000
Supercap	∼100%	>10,000	20∼40%	2.5∼15	10,000+	50,000+
Flywheel	>90%	1000∼5000	100%	10∼30	400∼1500	20,000+
CAES	∼70%	<50	Very low	5∼10 (Wh/L)	<1 (W/L)	—

Various research has studied the issue of charging strategies for ESDs. A model predictive control (MPC)-based charging strategy for Li-ion batteries is formulated in [23], based on a coupled electrothermal model for battery dynamics prediction. In the work [24], a convex programming (CP)-based control scheme is put forward to minimize the daily operational cost of a plug-in hybrid electric vehicle (PHEV). Studies have also been done about modeling for ESDs, such as comparison of equivalent circuit models for ultracapacitors in the context of driving-cycle-based loading, demonstrating that the dynamic model achieve the best overall performance [25], as well as fractional-order modeling to emulate the ultracapacitor dynamics by utilizing a series resistor, a constant-phase-element (CPE), and a Walburg-like element [26].

**Fig 1:**
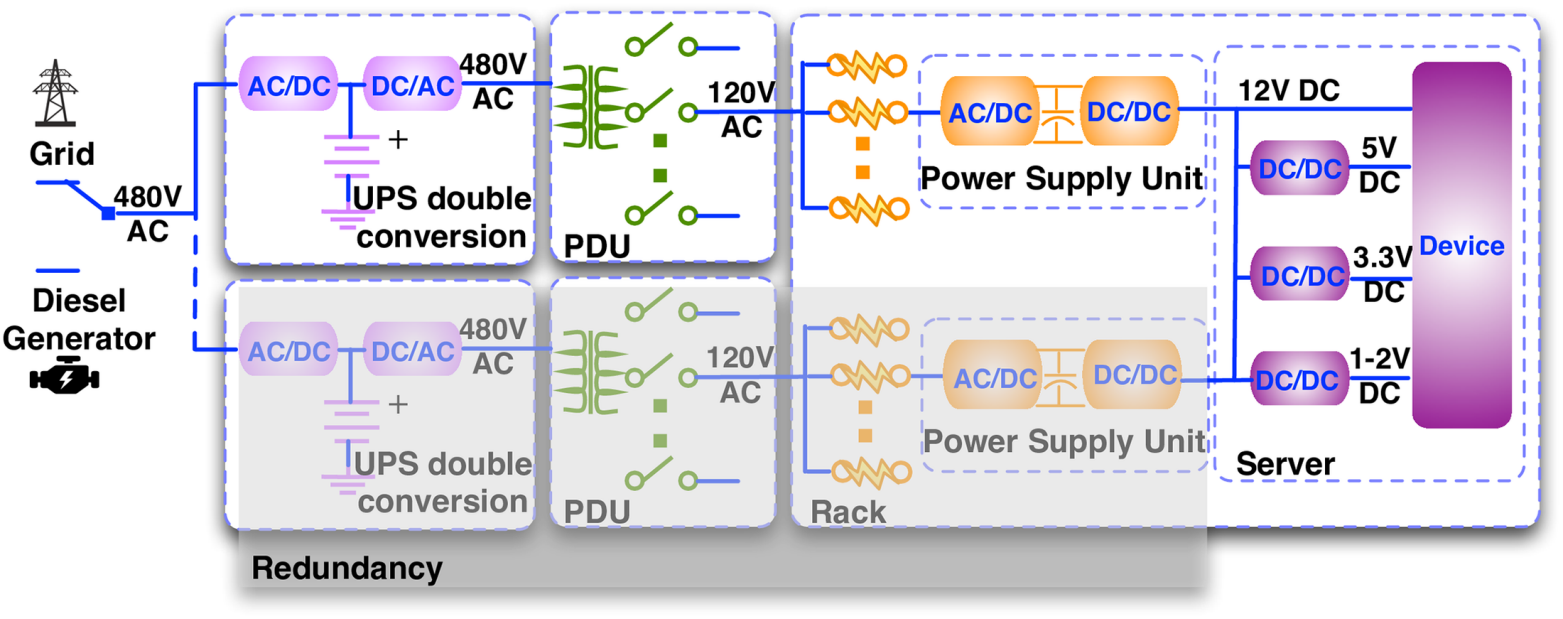
The power delivery architecture of a data center with the centralized ESD structure.

Traditional power delivery facilities in data centers adopt centralized ESD structure, with an example developed by Intel [27] shown in Fig 1. The 480V AC power from the utility grid or alternatively the diesel generator must first go through AC-DC-AC double conversion with the centralized ESD connected in between (because the ESD is essentially DC). This AC-DC-AC double conversion can guarantee seamless transition from the utility grid to ESDs and then to diesel generator, but it is the primary source of inefficiency in this design. A survey from Intel illustrated that the power delivery architecture may result in 20%-30% power loss [27], in which the AC-DC-AC double conversion may incur up to 15%. In the centralized ESD structure, a redundant UPS is required to improve the power reliability of data center in case one UPS malfunctions or temporarily shuts off for maintenance.

**Fig 2:**
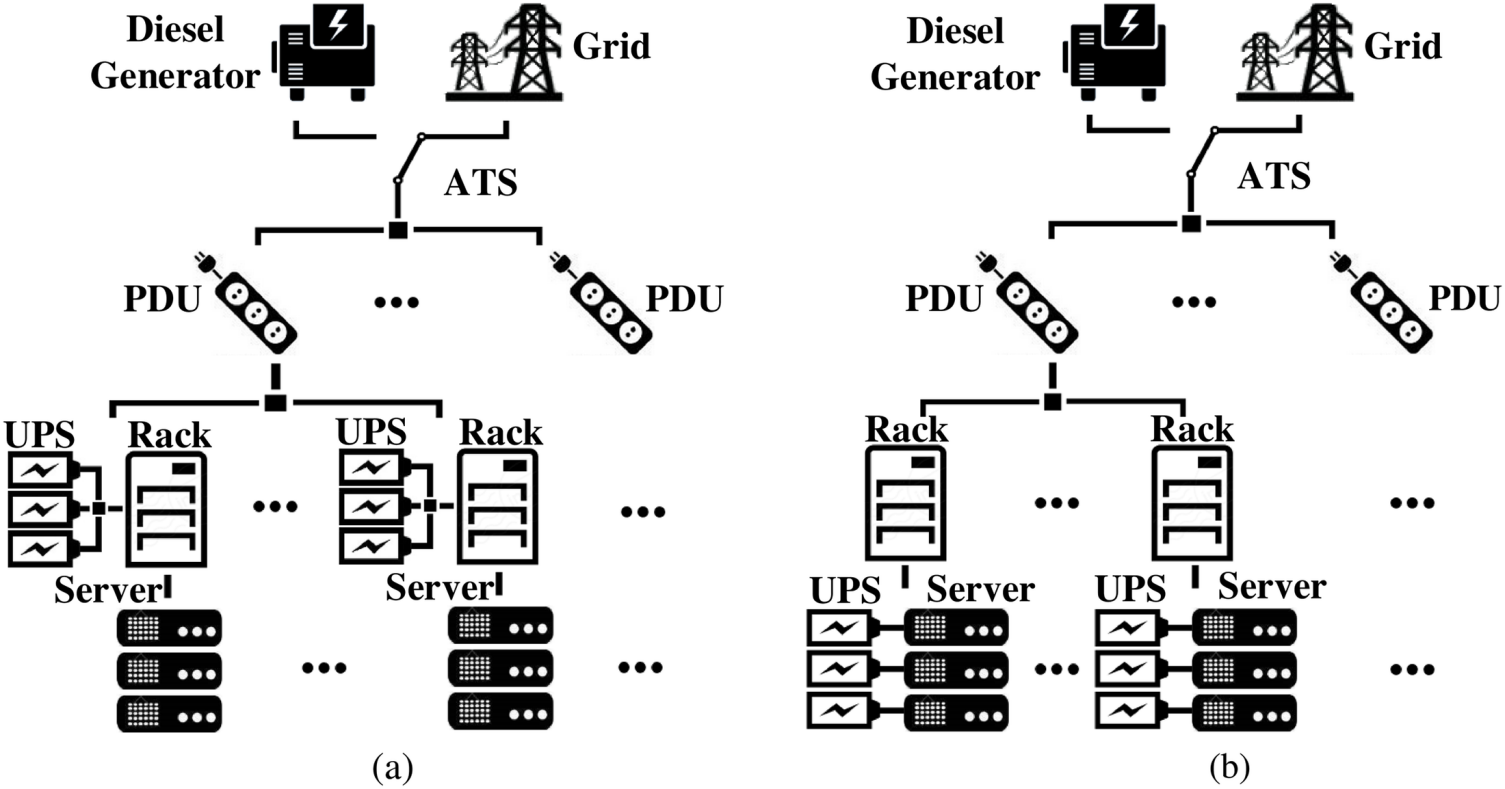
The power delivery architectures of a data center with the distributed single-level ESDs. (a) Architecture proposed by Microsoft. (b) Architecture proposed by Google.

Some state-of-the-art data centers by Google [28], Microsoft [29], and Facebook [30] employ the distributed single-level ESD structure, where ESDs are integrated into rack or server level and directly connected to the corresponding DC power buses. Fig 2(a) shows the distributed rack-level ESD structure from Microsoft [29], where ESDs are directly connected (without converters) to the rack-level DC bus, and Fig 2(b) gives the distributed server-level ESD structure from Google [28], where ESDs are directly connected to the server-level DC bus. Compared with the centralized counterpart, the distributed single-level ESD structure achieves less transmission power loss and thereby higher efficiency. Google reported that the achieved efficiency improvement corresponds to $30/server yearly cost saving [28]. However, the distributed single-level ESD structure may encounter serious volume/real-estate constraints since the space inside each rack is precious and limited, thereby restricting the ESD size and capability in performing power capping.

A hierarchical ESD structure could address this shortcoming by placing ESDs to data center, rack, and server levels, with the potential of taking advantages of both centralized and distributed ESD structures [3]. Moreover, proper deployment and control of multiple types of ESDs might achieve high capability in power capping and high energy capacity simultaneously [3, 21, 31, 32]. To fully realize the potential benefits of the hierarchical ESD structure, we propose a comprehensive design, control, and provisioning framework, including (i) designing the *power delivery architecture* (i.e., detailed and feasible connections of ESDs, power buses, power converters) supporting the hierarchical ESD structure and potentially hybrid ESDs (with more than one ESD type) for some levels, which is lacking in literature; (ii) control and provisioning of the hierarchical ESD structure including run-time ESD charging/discharging control and design-time determination of ESD type, homogeneous/hybrid options, ESD provisioning at each level. The technical contributions are summarized as follows:

1. Present high-efficiency power delivery architectures, supporting homogeneous ESD or hybrid ESD at each of data center, rack, and server levels;
2. Provide a scalable control framework to determine charging/discharging of various (homogeneous or hybrid) ESDs, to minimize capex and opex simultaneously.
3. Provide a scalable provisioning framework to determine appropriate ESD type, homogeneous/hybrid options, and ESD provisioning at each level. A comprehensive profit analysis is used to achieve high capability in capex and opex reductions, accounting for realistic characteristics such as ESD volume constraints for each level and capital costs, power rating of ESDs and power converters, etc;
4. Conduct extensive experiments using real Google data center workload traces based on realistic data center specifications.

## Materials and methods

### Power delivery architecture for hierarchical ESD structure

The proposed hierarchical ESD structures borrow the best features of centralized ESD structure from Intel [27] and distributed single-level ESD structures from Google [28] and Microsoft [29], therefore simultaneously mitigate efficiency (and redundancy) shortcomings of the centralized structure and the space limitation of distributed single-level structures.

The proposed *Hier-Homo* and *Hier-Hybrid* architectures have respective advantages and applicable conditions. The *Hier-Homo* architecture is relatively straightforward to implement and control, whereas the *Hier-Hybrid* is a more general architecture that can be reduced to *Hier-Homo* if only the primary ESDs are employed. The *Hier-Hybrid* architecture is more advanced to further reduce the cost, more complicated in control and provisioning, and therefore more suitable for large-scale data center systems.

#### The *Hier-Homo* architecture

**Fig 3:**
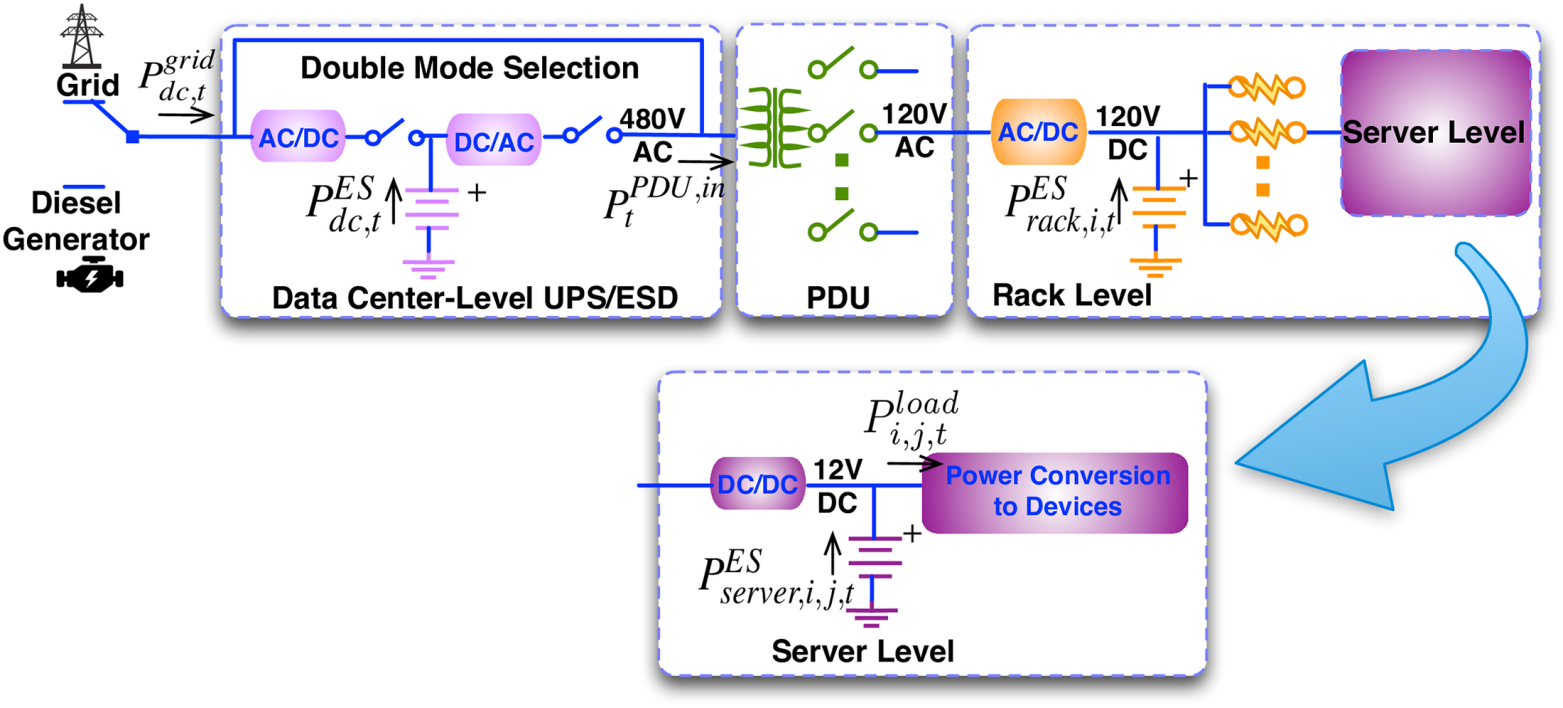
Illustration of the proposed *Hier-Homo* architecture.

Fig 3 displays the proposed *Hier-Homo* architecture. Please note that different types of ESDs can be employed at each of the three levels but for each level only homogeneous ESD can be supported. We employ a new type of data center-level UPS connection method presented in [18] that can be operated in either double-conversion mode or high-efficiency mode by effectively controlling a set of programmable switches. As reported in [18], the high efficiency mode, which bypasses input power from grid to PDU, improves the energy efficiency by up to 10% compared with the double-conversion mode. The time to switch between these two modes is only one AC cycle (16.7ms in a 60Hz grid), which can be handled by rack/server-level UPS or server exception handlers.

The *Hier-Homo* architecture comprises four stages: (i) 480V AC power from data center-level UPS connection. (ii) The PDU transforms 480V AC into 120V AC and distributes to each rack. (iii) The 120V AC power is first rectified into 120V DC through AC/DC rectifier and then distributed to each server inside the rack. Rack-level ESDs are directly connected to 120V DC buses without power converters. (iv) For each server, the 120V DC power is converted to 12V DC. Server-level ESDs are directly connected to 12V DC buses. Power conversion efficiencies of various converters will be similar to those results from [27] (because similar conversion circuitries are utilized) except for the data center-level connection in high efficiency mode, which is close to 100% (97% as reported in [18].)

The *Hier-Homo* architecture combines the advantages of both centralized and distributed single-level ESD structures while hiding their weaknesses. It avoids AC-DC-AC double conversion if the data center-level ESD/UPS is not in use (by switching to the high efficiency mode), and directly connects rack-level and server-level ESDs to corresponding DC buses, thereby significantly reducing the power losses. When power outage happens, the ESDs at each level can immediately provide backup power during the grid to diesel generator transition. High power supply reliability can be achieved without redundancies.

#### The *Hier-Hybrid* architecture

**Fig 4:**
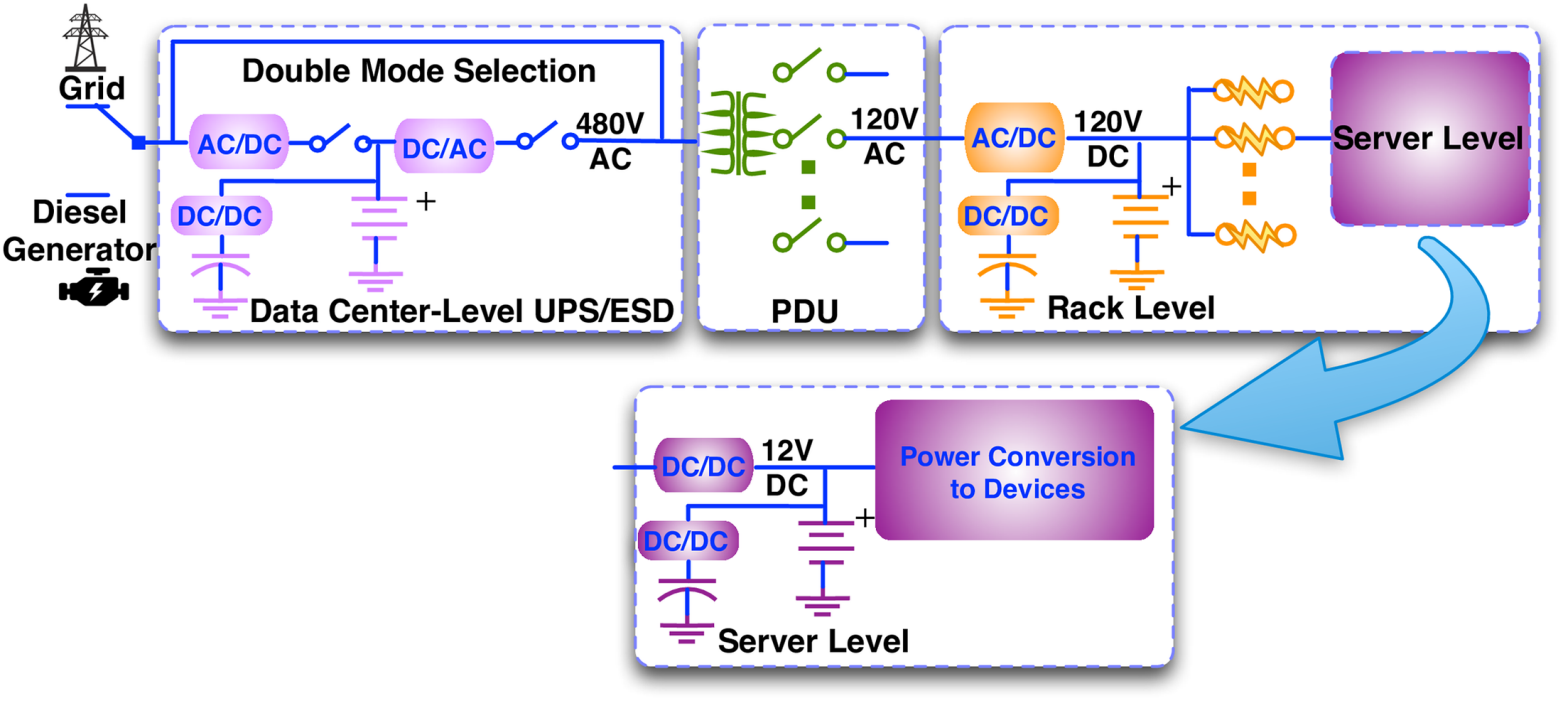
Illustration of the proposed *Hier-Hybrid* architecture.

Since different types of ESDs exhibit distinct characteristics such as energy density, power density, etc., it may be beneficial to incorporate hybrid ESDs to accommodate different power demands, e.g., utilizing supercapacitors for short-term high-peak power demands while deploying batteries to accommodate long-term and relatively low-peak power demands. Fig 4 illustrates the proposed *Hier-Hybrid* architecture, which accommodates hierarchical ESD structure and hybrid ESDs at potentially the data center, rack, and/or server levels. The *Hier-Hybrid* architecture is general and can accommodate other types of ESDs such as supercapacitors, flywheels, or CAES. We assume at most two types of ESDs (in the hybrid ESD) at each level due to complexity considerations, and different combinations of hybrid ESDs (e.g., lead-acid battery and Li-ion battery, Li-ion battery and supercapacitor) can be employed for each level.

The hybrid ESD at each level comprises of two parts: (i) an ESD bank with relatively stable terminal voltage, termed the *primary* ESD, which is directly connected to the DC bus (similar to the homogeneous ESDs in the *Hier-Home* architecture), (ii) an additional ESD bank, termed the *secondary* ESD, which is connected to the DC power bus through a bi-directional converter (or other power conversion and auxiliary circuitry if flywheels or CAES are adopted). The “bi-directional” property is necessary due to the requirement of charging and discharging of secondary ESD. Please note that the hybrid ESD is a general concept, in that it can possibly comprise only the primary ESD without secondary ESD (similar to the ESD in *Hier-Homo* architecture), or only the secondary ESD.

The *Hier-Hybrid* architecture also avoids AC-DC-AC double conversion if the data center-level ESD/UPS is not in use. The primary ESDs in the *Hier-Hybrid* architecture are also directly connected to the corresponding DC buses, similar to ESDs in the *Hier-Homo* architecture. However, the secondary ESDs are connected to the corresponding DC buses through a DC/DC converter, which will inevitably incur power loss. Therefore, the actual benefit for applying hybrid ESDs needs to be scrutinized when accounting for the more sophisticated power delivery architecture and control mechanisms.

### Control framework of hierarchical ESD structure

A scalable control framework is presented to determine ESD charging/discharging to achieve simultaneous minimization of capex and opex. Effective methods are utilized to significantly reduce the computation complexity and make the amount of computation scalable with the number of servers in a data center, which is critical for the (adaptive) run-time control requirement.

#### Hierarchical controller scheme for hierarchical ESDs

**Fig 5:**
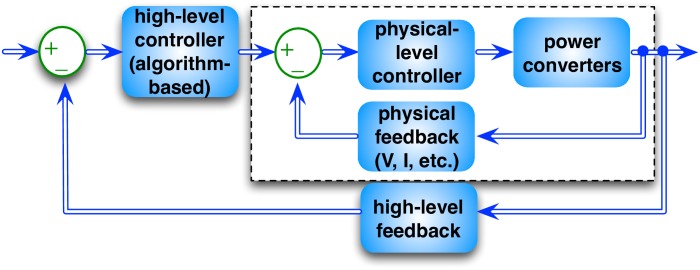
Hierarchical, closed-loop control for hierarchical ESD structure.

In general, supervisory control should be applied to manage the energy flow direction and the amount of energy transfer in the system. On the other hand, power converters can regulate their output voltage or current according to the control set points from supervisory controller. The proposed a hierarchical control scheme for hierarchical ESDs is illustrated in Fig 5. More precisely, physical-level controllers of power converters are adopted to regulate their output voltage/current and guarantee stability against external disturbances such as terminal voltage and temperature variations. The physical-level control loops will be implemented with hardwired controller for each power converter.

The regulation principle is explained using the *Hier-Homo* architecture. The server-level DC bus is 12V and is determined by terminal voltage of server-level ESD. The server-level converter regulates its output power through regulating output current. The output power of server-level ESD, denoted by $P_{server,i,j,t}^{ES}$ in Fig 3, is determined as the difference between the output power of server-level converter and load power demand. The rack level is similar. At the data center level, the set of programmable switches decide the operating mode: high efficiency mode or double-conversion mode. In the former mode, the AC-DC-AC double conversion will be bypassed. In the latter mode, the input power of the DC/AC inverter is decided by its output power $P_{t}^{\text{PDU},in}$ and the conversion efficiency. The AC/DC rectifier regulates its output power through regulating output current, and the output power of data center-level ESD, $P_{dc,t}^{ES}$, is decided accordingly.

For the *Hier-Hybrid* architecture, the basic regulation principle is similar to that for the *Hier-Homo* architecture, except for the difference in the control of hybrid ESDs. More specifically, for the hybrid ESDs at the rack level or the server level, the voltage level of the corresponding DC bus is determined by the terminal voltage of primary ESD. The output current (and power) of the secondary ESD is determined by the connected bi-directional DC/DC converter based on high-level control policy. Then the output power of the primary ESD is determined accordingly, based on the load power consumption (of server or rack), output power of the corresponding (AC/DC or DC/DC) converter, and output power of the secondary ESD.

#### High-level control algorithm for hierarchical ESDs

**Objective function.** The high-level control algorithm is adaptive and will be performed at each decision epoch during a billing period, which may range from an hour to multiple days. The main objective is to achieve simultaneous minimization of amortized capex and opex during this billing period, given by:

$$\begin{array}{c} \begin{array}{cc} & Amortized\_Infrastructure\_Capex+\\ & Amortized\_ESD\_Capex+Peak\_Power\_Tariff+\\ & Dynamic\_Energy\_Cost \end{array} \end{array}$$ (1)

The first two terms correspond to the amortized capex in this billing period. The first term is amortized capex of power infrastructure, often estimated at $10-20 per Watt [2]. The second term is amortized ESD degradation cost. When ESD ages, its capacity reduces and internal resistance increases [33]. When the ESD reaches its end-of-life, it needs to be replaced and will incur a high replacement cost. On the other hand, we consider a general opex comprised of both peak power tariff *Peak*_*Power*_*Tariff* (c.f. [34, 35]) and the cost component associated with the dynamic pricing policy (c.f. [34, 36]). *Peak*_*Power*_*Tariff* is given by:

$$\begin{array}{c} Peak\_Power\_Tariff=Peak\_Price\cdot \max_{t\in \text{billing}\;\text{period}}P_{dc,t}^{grid} \end{array}$$ (2)

where $P_{dc,t}^{grid}$ is the data center power consumption drawn from the grid in time slot *t*. *Dynamic*_*Energy*_*Cost* is calculated by:

$$\begin{array}{c} \begin{array}{cc} & Dynamic\_Energy\_Cost=\\ & \sum_{t\in \text{billing}\;\text{period}}Energy\_Price_{t}\cdot P_{dc,t}^{grid}\cdot \Delta \end{array} \end{array}$$ (3)

assuming time-ahead dynamic pricing (c.f. [34, 36, 37]) is used, where *Energy*_*Price*_*t*_ is dynamic energy price in time slot *t*, and Δ is slot duration.

The motivations and challenges of the control algorithm are as follows: (i) At each decision epoch, the control algorithm needs to derive the charging/discharging schedule of all data center-level, rack-level and server-level ESDs. It should have reasonable computational complexity for run-time implementation and be scalable with the number of servers, since modern data center comprises tens of thousands of servers [28, 29]; (ii) Data center-level ESDs should be avoided if possible to enhance the overall energy efficiency; (iii) The algorithm should account for energy loss in the ESD charging/discharging process and in the power converters in order to achieve optimal power capping; (iv) Different aging rates of different types of ESDs should be accommodated when determining the charging/discharging power of the primary and secondary ESDs to reduce the amortized ESD capex.

A heuristic algorithm is brought forward for data center hierarchical ESD control to tackle the above-mentioned challenges. Executed at each decision epoch, the control algorithm consists of two steps, the first determining whether to charge or discharge these hierarchical ESDs, and the second deciding the detailed charging/discharging schedule of the ESDs.

**Step 1.** Whether to charge or discharge the hierarchical ESDs is decided by comparing the current power consumption of the data center, denoted by $P_{dc}^{total}$, with the peak power consumption of the data center in the previous billing period, denoted by $P_{dc}^{grid,peak}$. If $P_{dc}^{total}$ is larger than $P_{dc}^{grid,peak}$, the ESDs should be discharged for power capping unless the energy stored in the ESDs is not sufficiently high. Otherwise, the ESDs should be charged. The value of $P_{dc}^{grid,peak}$ could be dynamically updated for the effectiveness of the control algorithm. $P_{dc}^{grid,peak}$ is used as a criterion for two reasons. One is that the infrastructure capex is directly proportional to data center’s peak power consumption [2]. The other is that for the opex, the peak power tariff component, given by Eq (2), is more prominent than the dynamic energy cost component, given by Eq (3).

**Step 2.** The detailed charging/discharging schedule of the ESDs at the current time slot is generated by a heuristic, *budget allocation-based* method. Specifically, the scheduling procedure is composed of three operations, i.e., (i) budget allocation to calculate the power demand (e.g., $P_{server,i,j,t}^{total}$) of ESDs, (ii) deriving the actual charging/discharging power (e.g., $P_{server,i,j,t}^{ES}$) of ESDs by incorporating the efficiency of power converters, and (iii) applying a crossover filter method to determine the actual charging/discharging power of each primary and secondary ESDs. Without loss of generality, the more general *Hier-Hybrid* architecture is used to explain this budget allocation method, which is also applicable to the *Hier-Homo* architecture.

The details of the three operations are as follows. (i) If the ESDs need to be discharged, it is necessary to determine whether the data center-level ESDs would be discharged or not, since, as discussed before, the energy efficiency would be low when utilizing data center-level ESDs as the energy flow goes through more power converters. Therefore, if the difference between $P_{dc}^{grid,peak}$ and $P_{dc}^{total}$ is smaller than a threshold value, then only the rack-level and server-level ESDs are planned to be discharged, to guarantee that the ESDs are not over-discharged, and the overall energy efficiency of discharging can be relatively high. Otherwise, the data center-level ESDs should also be discharged to provide sufficient total discharge power. In the situation where only the rack and server-level ESDs are discharged, the power provided from the ESDs is allocated according to their stored energy. The total energy stored in the rack-level ESDs of the *i*-th rack is denoted by $E_{i,t}^{rack}$, and comprises the energy stored in both the primary ESD and the secondary ESD. $E_{i,j,t}^{server}$ denotes the total energy stored in the server-level ESDs of the *j*-th server in the *i*-th rack. The power allocation of the server-level ESDs of the *j*-th server in the *i*-th rack is then calculated by

$$\begin{array}{c} \begin{array}{cc} & P_{server,i,j,t}^{total}=\frac{E_{i,j,t}^{server}}{\sum_{i=1}^{N}E_{i,t}^{rack}+\sum_{i=1}^{N}\sum_{j=1}^{M_{i}}E_{i,j,t}^{server}}\\ & \cdot (P_{dc}^{total}-P_{dc}^{grid,peak}) \end{array} \end{array}$$ (4)

where *N* is the number of racks, and *M*_*i*_ is the number of servers in the *i*-th rack. Similarly, the power allocation of the rack-level ESDs of the *i*-th rack is calculated by

$$\begin{array}{c} \begin{array}{cc} & P_{rack,i,t}^{total}=\frac{E_{i,t}^{rack}}{\sum_{i=1}^{N}E_{i,t}^{rack}+\sum_{i=1}^{N}\sum_{j=1}^{M_{i}}E_{i,j,t}^{server}}\\ & \cdot (P_{dc}^{total}-P_{dc}^{grid,peak}) \end{array} \end{array}$$ (5)

(ii) According to the power allocation of the server-level ESDs, i.e., $P_{server,i,j,t}^{total}$, the total discharge power of the server-level ESDs, denoted by $P_{server,i,j,t}^{ES}$, can be derived by incorporating the efficiency of power converters along the energy flow. $P_{server,i,j,t}^{ES}$ is higher than $P_{server,i,j,t}^{total}$ because of the energy loss of the power converters. (iii) The crossover filter method is then applied in [38] to derive the discharge power of the primary and secondary ESDs, with the purpose of slowing down the ESD aging and thereby reducing the amortized ESD capex. For example, when the primary ESD is Li-ion battery and the secondary ESD is supercapacitor, the filtering technique allows the battery to stably receive/provide power while leaving the spiky charging/discharging power to be dealt with by the supercapacitor, and thereby prolonging the overall cycle life of the ESDs. The same approach is employed to derive the discharge power of the primary and secondary ESDs at rack-level, given the $P_{rack,i,t}^{total}$ value.

In the situation where the data center-level ESDs should also be discharged, the budget allocation method is applied for all the data center-level, rack-level, and server-level ESDs according to the corresponding stored energy. Similarly, if the ESDs need to be charged, budget allocation can be applied to derive the charging power of each ESD. In this case budget allocation is based on the amount of energy so that each ESD can be charged to 100% state-of-charge. The pseudocode of the control algorithm is given in Algorithm 1. Overall, the proposed control algorithm exhibits low computational complexity and the associated computation requirements are negligible compared with the whole data center, which typically consumes MWs of power consumption.

**Algorithm 1** The hierarchical ESD control algorithm

**Require:** the system architecture, the energy stored in each ESD, peak power consumption in previous billing period i.e., $P_{dc}^{grid,peak}$, the current power consumption of the data center when ESDs are not used i.e., $P_{dc}^{total}$

**Ensure:** the charging/discharging power of each ESD

 **if** $P_{dc}^{total}>P_{dc}^{grid,peak}$ **then**

  **if** $P_{dc}^{total}-P_{dc}^{grid,peak}<\Delta_{th}$ **then**

   %Discharge rack and server-level ESDs.

   Budget allocation to determine $P_{rack,i,t}^{total}$ and $P_{server,i,j,t}^{total}$.

   Derive $P_{rack,i,t}^{ES}$ and $P_{server,i,j,t}^{ES}$ according to system architecture.

   Crossover filter to determine the discharge power of primary ESDs and secondary ESDs.

  **else**

   %Discharge data center, rack and server-level ESDs.

   Budget allocation to determine $P_{dc,t}^{total}$, $P_{rack,i,t}^{total}$ and $P_{server,i,j,t}^{total}$.

   Derive $P_{dc,t}^{ES}$, $P_{rack,i,t}^{ES}$ and $P_{server,i,j,t}^{ES}$ according to system architecture.

   Crossover filter to determine the discharge power of primary ESDs and secondary ESDs.

  **end if**

 **else**

  %Charge data center, rack and server-level ESDs.

  Budget allocation to determine $P_{dc,t}^{total}$, $P_{rack,i,t}^{total}$ and $P_{server,i,j,t}^{total}$.

  Derive $P_{dc,t}^{ES}$, $P_{rack,i,t}^{ES}$ and $P_{server,i,j,t}^{ES}$ according to system architecture.

  Crossover filter to determine the charge power of primary ESDs and secondary ESDs.

 **end if**

### Provisioning framework of hierarchical ESD structure

The provisioning framework is presented to determine the appropriate ESD types, homogeneous/hybrid options, and ESD capacities at each level, in order to minimize overall capex and opex using a comprehensive profit analysis. This framework will account for realistic aspects such as ESD volume constraints (for each level) and capital costs of ESDs, power rating of ESDs and power converters, etc. Effective methods are utilized to significantly reduce the computation complexity for the provisioning framework and make it efficient for design-time optimizations. The more general *Hier-Hybrid* architecture is used to demonstrate the provisioning framework, which can also be applied to the *Hier-Homo* architecture.

The provisioning framework optimizes the types and energy capacities of primary and secondary ESDs at different levels, i.e., $Type_{dc}^{pri}$, $Type_{dc}^{sec}$, $Type_{rack}^{pri}$, $Type_{rack}^{sec}$, $Type_{server}^{pri}$, $Type_{server}^{sec}$, and $E_{dc}^{C,pri}$, $E_{dc}^{C,sec}$, $E_{rack}^{C,pri}$, $E_{rack}^{C,sec}$, $E_{server}^{C,pri}$, $E_{server}^{C,sec}$. Please note that the type of the secondary ESD could be “N/A” to denote homogeneous ESD at that level. A straightforward search-based algorithm would search all the possible values of the 12 variables and result in a prohibitive computational complexity of *O*(*T*^6^ ⋅ *S*^6^), where *T* is the number of available ESD type options and *S* is the search precision level of the energy capacities.

**Fig 6:**
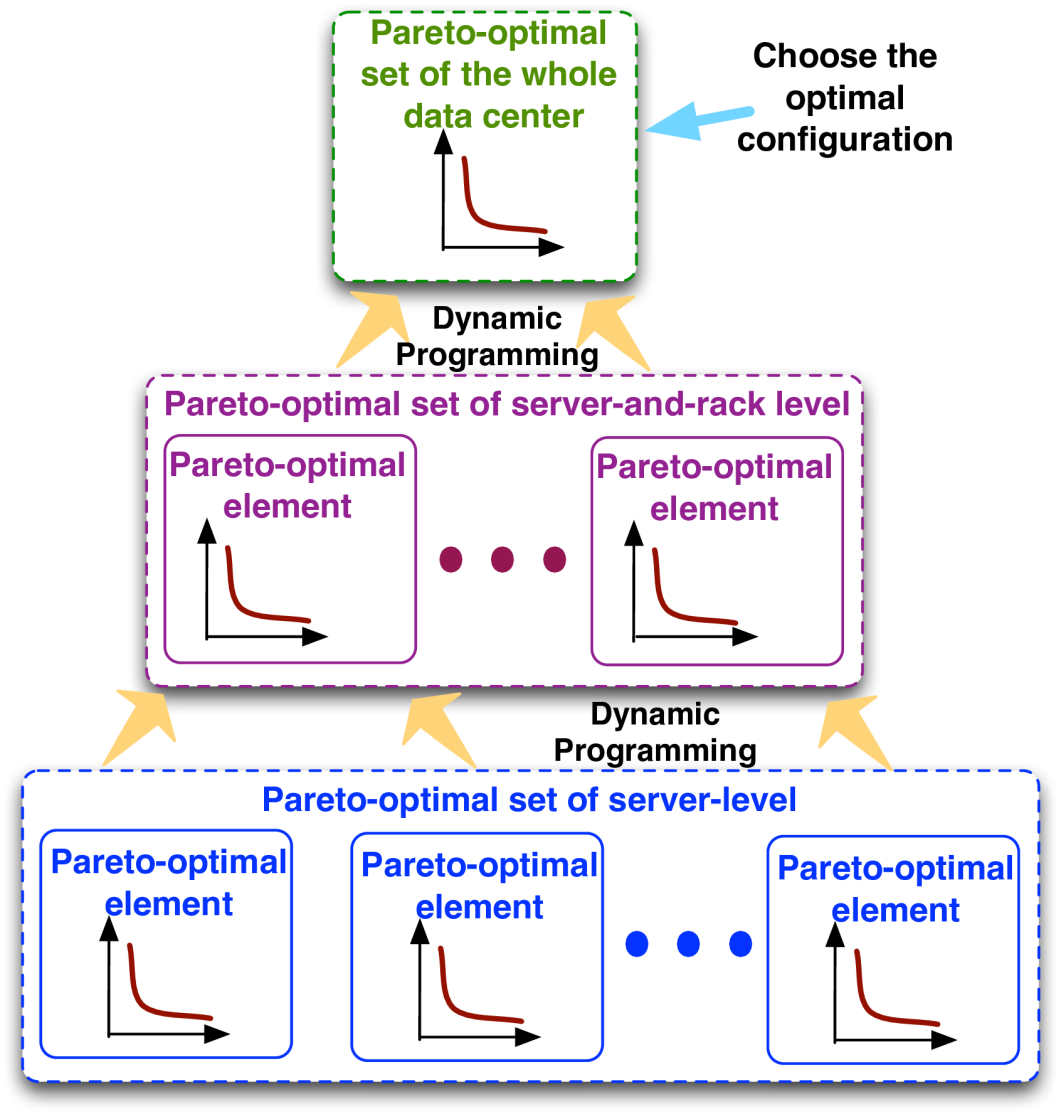
The provisioning framework of the *Hier-Hybrid* architecture.

An effective and efficient provisioning algorithm is brought forward, as shown in Fig 6 to reduce complexity using the idea of *dynamic programming based on Pareto-optimal sets* [39]. The first step is to derive the Pareto-optimal set of the type and energy capacity combinations of the primary and secondary ESDs for the server level. For example, an element in the Pareto-optimal set of the server-level is $\left[Type_{server}^{pri},Type_{server}^{sec},E_{server}^{C,pri},E_{server}^{C,sec}\right]$. Each element in the derived Pareto-optimal set is Pareto-optimal with respect to the power capping capability (in terms of capex and opex reduction) and capital cost. This derivation procedure accounts for various realistic constraints for the servers and is based on detailed profit analysis under realistic data center specifications and pricing policies.

In the next step, the dynamic programming algorithm [40] is employed to derive the Pareto-optimal set of the server-and-rack level (two levels) based on the previously derived server-level Pareto-optimal set. Subsequently, the final Pareto-optimal set of all the ESDs in the data center is constructed with the server-and-rack-level Pareto-optimal set and the dynamic programming algorithm. This procedure has polynomial time complexity and reasonable computing time for design-time optimization, because the employment of Pareto-optimal set excludes many infeasible solutions at the early stage. Finally the optimal choice among all elements in the final Pareto-optimal set in terms of overall capex and opex reduction satisfying the overall capital cost constraint, and the corresponding ESD types, homogeneous/hybrid options, and ESD capacities for all the levels.

## Results and discussion

Experimental results are provided for both the control and provisioning frameworks of the *Hier-Homo* and *Hier-Hybrid* architectures. A realistic data center setup similar to that in [3] is considered, comprising 8,192 servers placed in 256 racks with 32 servers/rack, and exhibits 4 MW peak power consumption. The data center is equipped with the *Hier-Homo* or *Hier-Hybrid* architectures as discussed above. The billing period is set to be one day and time slot Δ_*t*_ = 5 minutes. Google cluster trace is used as realistic workloads for our evaluations [41]. The Google cluster trace released in 2012 is measured in a 29-day period including 627,075 jobs and more than 48 million tasks. The (normalized values of) CPU, memory, and disk utilizations of the server cluster are measured and recorded for every 5 minutes. The load power consumption $P_{i,j,t}^{load}$ of each server is derived based on CPU and memory utilization traces and accurate server power modeling [42].

**Fig 7:**
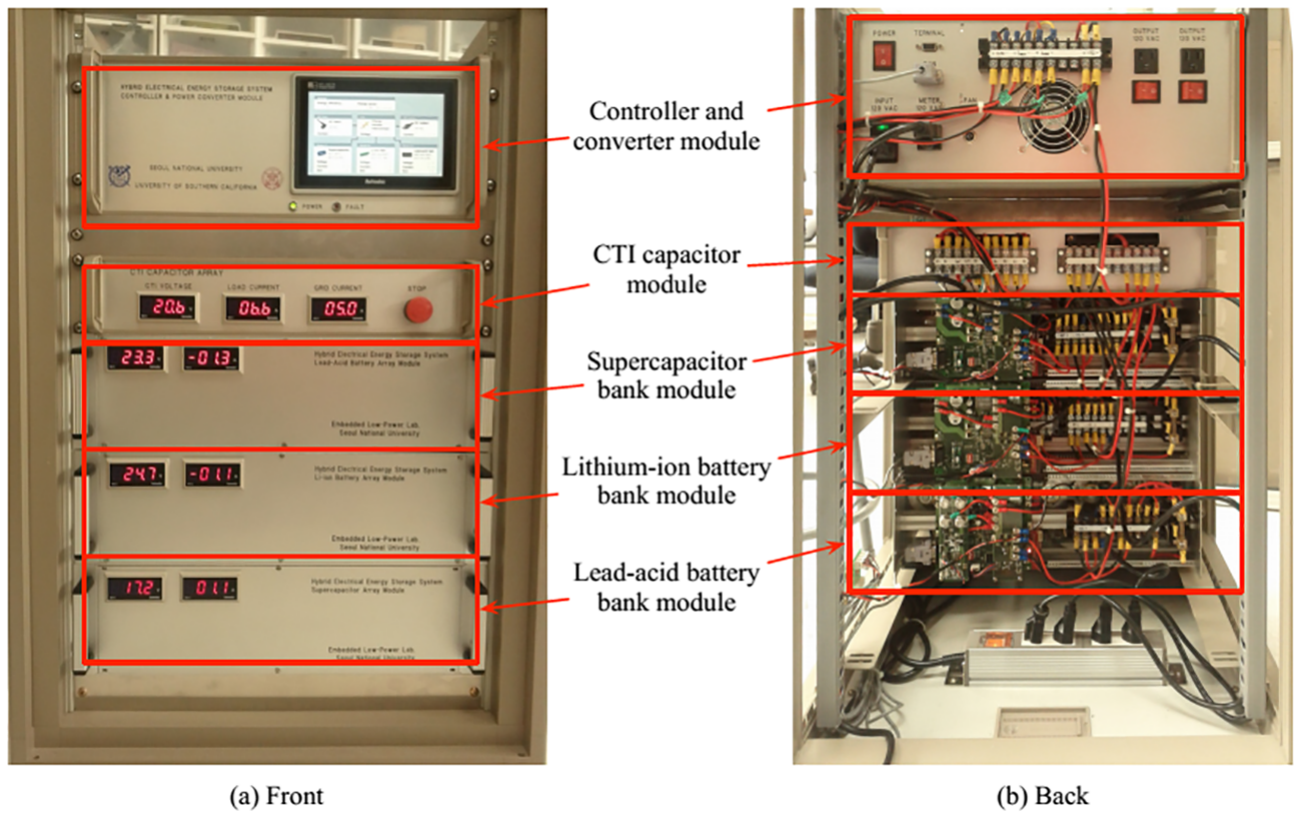
Our implementation of a hybrid ESD system.

For the *Amortized*_*Infrastructure*_*Capex* component in the objective function, the parameter is set to be $15 per Watt of peak power [2], and the total infrastructure capex is amortized over 20 years. For the opex, a realistic dynamic energy pricing policy similar to the one of LADWP [34] is adopted, consisting of a time-of-day energy price component and a peak price component (peak power tariff). The energy price component is given by: 0.01879 $/kWh during 00:00 to 09:59 and 20:00 to 23:59, 0.03952 $/kWh during 10:00 to 12:59 and 17:00 to 19:59, 0.04679 $/kWh during 13:00 to 16:59. The peak price component is given by 0.575 $/kW to charge the peak power consumption over the whole day. A hybrid ESD system has been implemented, as shown in Fig 7 consisting of Li-ion batteries, lead-acid batteries, supercapacitors and power converters to measure the related parameters of ESDs and power converters. For the parameters of flywheels and CAES, the related parameters are imported from [21, 22].

### Results of the *Hier-Homo* architecture

Two *Hier-Homo* ESD architectures are first considered, one only using lead-acid batteries and the other only using Li-ion batteries. For both (lead-acid based and Li-ion based) architectures, 0.24kWh is used as the energy capacity for each server-level ESD, which corresponds to 3L in volume for lead-acid batteries when assuming energy density of 80kWh/m^3^, or 0.75L in volume for Li-ion batteries when assuming energy density of 320kWh/m^3^. Let 32×0.24kWh be the energy capacity for each rack-level ESD for both architectures, where 32 is the number of servers in a rack. Then, let 8192×0.24kWh be the energy capacity for data center-level ESD for both architectures, where 8192 is the number of servers in a data center. The volume limits for server and rack-level ESDs are assumed to be 3L, 32×3L, respectively. The volume constraint for the data center-level ESDs is not set, since a dedicated building or space can be used to accommodate those ESDs.

The two proposed systems are compared with three baseline systems. Baseline 1 adopts the lead-acid battery-based centralized ESD structure used in [18] and the ESD/UPS is not utilized for power capping. Baseline 1 serves as the basis for our comparisons, because the state-of-the-art ESD/UPS in data centers are made of lead-acid batteries and are not yet utilized for power capping. Baseline 2 also uses lead-acid battery-based centralized ESD structure and the ESD/UPS is controlled for capex and opex reduction and power capping. The energy capacity of the ESD in Baseline 2 equals to the summation of energy capacities of all ESDs in the proposed system. Baseline 3 utilizes Li-ion battery-based centralized ESD structure and the ESD/UPS is controlled for capex and opex reduction and power capping. The energy capacity of the ESD in Baseline 3 equals to the summation of energy capacities of all ESDs in the proposed system.

**Fig 8:**
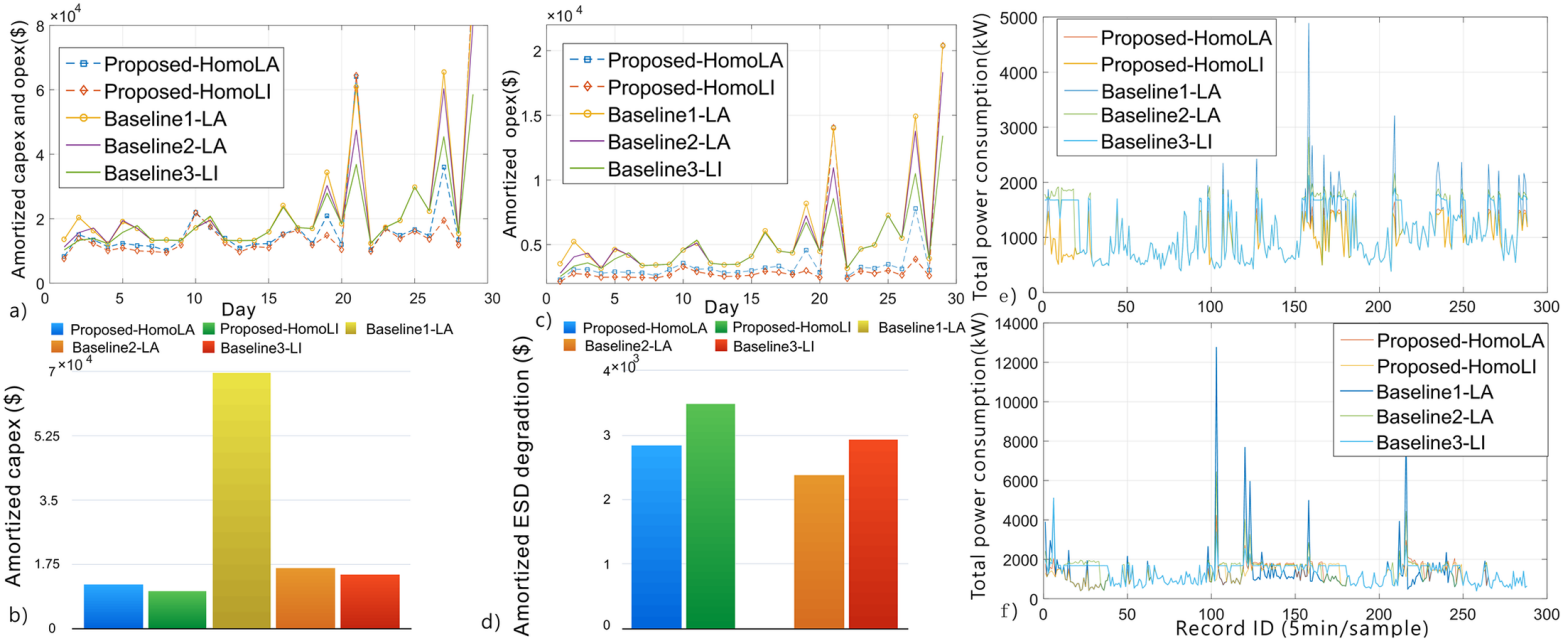
Comparison results. (a) Amortized capex and opex i.e., objective function. (b) Amortized infrastructure capex. (c) Daily opex. (d) Amortized ESD capex of the two proposed systems and baseline systems. (e)-(f) The data center power consumption drawn from grid, i.e., $P_{dc,t}^{grid}$, demonstrating the power capping capability.

Fig 8 shows comparisons of the two proposed systems and baseline systems. More specifically, Fig 8(a) provides the (daily) amortized total cost. It is clear that the two proposed systems significantly outperform the three baselines, with a maximum reduction in the amortized total cost of 70.31%. These results demonstrate the higher performance in capex and opex reduction of the hierarchical ESD structure compared with the centralized structure with the same total ESD capacity. Between the two proposed systems, the Li-ion battery-based system outperforms the lead-acid battery-based one due to its higher efficiency and longer cycle life. Among all baselines, Baseline 1 performs the worst since it uses the least optimized structure (centralized ESD structure) and does not use ESDs for cost reduction and power capping, while Baseline 3 performs the best since it adopts Li-ion batteries and performs optimization for cost reduction and power capping. More detailed comparisons are provided in Fig 8(b) on the amortized infrastructure capex, (c) on the daily opex, and (d) on the amortized ESD degradation cost. It can be observed that (i) the proposed systems significantly outperform baselines in terms of amortized infrastructure capex reduction (due to the control optimization), although they may result in higher ESD degradation cost compared with baselines (due to the more often charging/discharging of ESDs); (ii) the Li-ion battery based system outperforms the lead-acid battery based system in capex reduction (due to the higher efficiency of Li-ion batteries) while it only incurs slightly higher ESD degradation (although Li-ion batteries have higher unit capital cost compared with lead-acid batteries, they also have longer cycle life.) Finally, Fig 8(e) and 8(f) give the data center power consumption drawn from the grid, i.e., $P_{dc,t}^{grid}$, demonstrating the power capping capability of the proposed systems and baselines.

Furthermore, different *Hier-Homo* architectures are compared with different ESD types at the data center, rack, and server levels. The ESD energy capacities at the data center, rack, and server levels are 8192×0.24kWh, 32×0.24kWh, and 0.24kWh, respectively. As discussed before, only lead-acid batteries and Li-ion batteries can be used for the *Hier-Homo* architecture, due to their relatively stable terminal voltages. The comparisons on average total cost (objective function), amortized infrastructure cost, amortized ESD capex, and average opex are shown in Table 2, from which it can be observed that under the setup of same ESD energy capacities in the *Hier-Homo* architecture, the all Li-ion battery system achieves the lowest amortized total cost, the lowest amortized infrastructure cost, the lowest opex, but relatively higher amortized ESD degradation cost. This is due to the fact that the high energy efficiency and long cycle life of Li-ion battery outweigh its high unit capital cost, demonstrating the superiority of Li-ion battery as ESDs for data center usage.

**Table 2:** Comparisons of *Hier-Homo* architectures with different ESD type combinations.

data center-rack-server ESD type	avg. total cost (k$)	amor. infra. capex (k$)	amor. ESD capex (k$)	avg. opex (k$)
Lead-Lead-Lead	19.083	12.050	2.84	4.193
Lead-Lead-Li	17.869	10.451	3.42	3.998
Lead-Li-Lead	17.502	10.336	3.45	3.716
Lead-Li-Li	17.530	10.342	3.47	3.718
Li-Lead-Lead	17.688	10.353	3.47	3.865
Li-Lead-Li	17.517	10.333	3.41	3.774
Li-Li-Lead	17.512	10.335	3.47	3.707
Li-Li-Li	17.120	10.310	3.38	3.430

Then we solve the provisioning problem for the *Hier-Homo* architecture. The volume limits for server and rack-level ESDs are supposed to be 3L, 32×3L, respectively. By applying the provisioning framework, the optimal design for the *Hier-Homo* architecture is found to consist of 0.06kWh Li-ion battery for server-level ESD, 32×0.18kWh Li-ion battery for rack-level ESD, and 8192×0.24kWh Li-ion battery for data center-level ESD, with the amortized total cost (capex and opex) of 16.29k$. It can be obtained that (i) the all Li-ion battery system is the optimal, which coincides with the previous observations; (ii) the volumes of the Li-ion batteries in the optimal design are smaller than the corresponding volume limits, as the increase of amortized ESD degradation cost outweights the margin of capex and opex reductions with larger ESDs; (iii) the optimal structure uses all of data center-level, rack-level, and server-level ESDs, thereby achieving a desirable tradeoff between the higher efficiency of rack and server-level ESDs and the higher flexibility (to support the whole data center) of data center-level ESDs.

### Results of the *Hier-Hybrid* architecture

For the *Hier-Hybrid* architecture, the ESDs in the optimal *Homo-Hybrid* design in the previous section are considered as the primary ESDs, based on which the secondary ESDs of each type are added and analyzed. The effect of supercapacitors as the secondary ESDs is first investigated with different volumes at different levels. As discussed before, supercapacitors can be integrated into any of the server, rack, and data center levels as secondary ESDs. The comparison results are shown in Table 3. When performing the provisioning framework, we can observe that 16.19k$ is the minimum amortized total cost that one can achieve in the setup with the size of 8192×2L at data center-level. The results indicate that integration of supercapacitors as the secondary ESDs can indeed decrease the capex and opex, resulting from the very high energy (cycle) efficiency and long cycle life of supercapacitors. However, because of the high unit capital cost, the reduction in amortized total cost is relatively insignificant (the maximum reduction is from 16.29k$ in the optimal *Hier-Homo* design to 16.19k$), which makes the supercapacitor an unnecessary secondary ESD.

**Table 3:** Comparisons of different *Hier-Hybrid* architectures with supercapacitor as the secondary ESD at different levels, based on the optimal *Hier-Homo* architecture.

supercap level	supercap size	avg. total cost (k$)	amor. infra. capex (k$)	amor. ESD capex (k$)	avg. opex (k$)
server	1L	16.263	10.376	2.159	3.727
server	2L	16.266	10.340	2.209	3.717
rack	32×1L	16.261	10.369	2.167	3.725
rack	32×2L	16.291	10.362	2.206	3.722
data center	8192×1L	16.228	10.373	2.129	3.725
data center	8192×2L	16.190	10.325	2.152	3.713

Furthermore, flywheels and CAES are investigated as the secondary ESDs with different volumes at the data center level, similarly on the basis of the above-mentioned optimal *Hier-Homo* design. As stated previously, flywheels and CAES are only suitable for data center-level ESDs because they use other forms of energy storage and require relatively large power conversion circuitries. The comparison results are shown in Table 4, from which it can be obtained that both flywheels and CAES can reduce the capex and opex as secondary ESDs at data center-level. The lowest average total cost for the *Hier-Hybrid* architecture is 15.41k$, achieved with flywheels of size 8192×5L as the secondary ESDs. Based on the best results for the *Hier-Homo* architecture in Table 2 with Li-ion batteries as the primary ESDs at each level, the amortized infrastructure capex and average opex change slightly, respectively from 10.31k$ to 10.12k$ and from 3.43k$ to 3.65k$, while the amortized ESD capex decreased by 51% from 3.38k$ to 1.64k$.

**Table 4:** Comparisons of different *xHier-Hybrid* architectures with flyweel or CAES as secondary ESD at the data center-level, based on the optimal *Hier-Homo* architecture.

type	size	avg. total cost (k$)	amor. infra. capex (k$)	amor. ESD capex (k$)	avg. opex (k$)
flywheel	8192×1L	16.098	10.343	2.036	3.718
flywheel	8192×2L	15.906	10.273	1.934	3.698
flywheel	8192×5L	15.407	10.115	1.637	3.654
CAES	8192×1L	16.220	10.388	2.102	3.730
CAES	8192×2L	16.170	10.368	2.078	3.724
CAES	8192×5L	16.053	10.325	2.015	3.713
CAES	8192×10L	15.865	10.262	1.909	3.694

Overall, among all the three possible types of secondary ESDs (flywheels, supercapacitors, and CAES) with the same size at data center-level, flywheels are the most effective, with amortized infrastructure capex, amortized ESD capex, average opex, and average total cost all being the lowest. In some situations the average total cost for flywheels is even lower than that for CAES with a bigger size (for instance, comparing the optimal design with flywheels of size 8192×5L with a cost of 15.41k$ to the design with CAES of size 8192×10L with a cost of 15.87k$). The main reason is that flywheels have a moderate unit capital cost and meanwhile a very long cycle life. On the other hand, CAES has a too low energy density and therefore requires a extremely large space. Although flywheels exhibit significant self-discharge and cannot perform as primary ESDs, they can perform well as secondary ESDs like energy buffers, in which situation self-discharge will not have a severe effect.

## Conclusions

State-of-the-art data centers utilize centralized or distributed single-level ESD structure that suffers from either low energy efficiency due to AC-DC-AC double conversion structure, or serious volume/real-estate constraints for over-provisioning and performing power capping. A hierarchical ESD structure is proposed to handle this problem by placing ESDs to data center, rack, and server levels, taking advantages of both centralized and distributed ESD structures. Furthermore, a comprehensive design, control, and provisioning framework is presented, including (i) designing power delivery architecture supporting hierarchical ESD structure and hybrid ESDs for some levels, as well as (ii) control and provisioning of the hierarchical ESD structure including run-time ESD charging/discharging control and design-time determination of ESD types, homogeneous/hybrid options, ESD provisioning at each level. This framework accounts for constraints on ESD volume and the overall capital cost for ESDs, and power losses due to the rate capacity effect and conversion circuitry. Experiments have been conducted using real Google data center workloads based on realistic data center specifications, demonstrating the effectiveness of the proposed design, control, and provisioning framework. For the Hier-Homo ESD structure, various combinations of batteries at all levels are tested, and the most suitable design is composed of Li-ion batteries at all the server, rack and data center levels with respective energy capacities of 0.06kWh, 32×0.18kWh and 8192×0.24kWh for our simulation setup, and can reach an amortized total cost of 16.29k$, reducing by 70.31% compared to the baselines. For the Hier-Hybrid structure, three types of secondary ESDs are investigated to improve the performance based on the Hier-Homo design. The best results for the three types are all found when they are integrated into the data center-level. Specifically, the lowest cost is 16.19k$ for supercapacitors with capacity of 8192×2L, 15.41k$ for flywheels with capacity of 8192×5L, and 15.87k$ for CAES with capacity of 8192×10L, respectively. Therefore, using flywheels as the secondary ESDs is considered as the most suitable choice.

Potential future research may focus on further optimization for power delivery network, including the optimization of power converters (e.g., size of power switches and other L, C elements) based on data center specifications and realistic workloads, as well as the application of bi-directional AC/DC rectifiers at the rack-level or bi-directional DC/DC converters at the server-level to allow more flexible energy flow across multiple racks or servers. Another issue worth studying could be the applicability of the proposed Hier-Homo and Hier-Hybrid architectures for various data center systems.

## References

[1] Barroso LA, Clidaras J, Hölzle U. The datacenter as a computer: An introduction to the design of warehouse-scale machines. Synthesis lectures on computer architecture. 2013; 8(3): 1–154. doi: 10.2200/S00516ED2V01Y201306CAC024

[2] Kontorinis V, Zhang LE, Aksanli B, Homayoun JSH, Pettis E, Tullsen DM, et al. Managing Distributed UPS Energy for Effective Power Capping in Data Centers. Proc of the ACM International Symposium on Computer Architecture (ISCA); Portland, USA. 2012. p. 488–499.

[3] Wang D, Ren C, Sivasubramaniam A, Urgaonkar B, Fathy H. Energy Storage in Datacenters: What, Where, and How much? Proc of the ACM International Conference on Measurement and Modeling of Computer Systems (SIGMETRICS); London, UK. 2012. p. 187–198.

[4] Fan X, Weber WD, Barroso LA. Power Provisioning for a Warehouse-sized Computer. Proc of the ACM International Symposium on Computer Architecture (ISCA); San Diego, USA. 2007. p. 13–23.

[5] Govindan S, Wang D, Sivasubramaniam A, Urgaonkar B. Leveraging Stored Energy for Handling Power Emergencies in Aggressively Provisioned Datacenters. Proc of the ACM Architectural Support for Programming Languages and Operating Systems (ASPLOS); London, UK. 2012. p. 75–86.

[6] Cochran R, Hankendi C, Coskun AK, Reda S. Pack & Cap: Adaptive DVFS and Thread Packing Under Power Caps. Proc of the IEEE/ACM International Symposium on Microarchitecture (MICRO); Porto Alegre, Brazil. 2011. p. 175–185.

[7] Gandhi A, Harchol-Balter M, Das R, Lefurgy C. Optimal Power Allocation in Server Farms. ACM SIGMETRICS Performance Evaluation Review. 2009; 37: 157–168.

[8] Gandhi A, Gupta V, Harchol-Balter M, Kozuch MA. Optimality Analysis of Energy-Performance Trade-off for Server Farm Management. Performance Evaluation. 2010; 67: 1155–1171. doi: 10.1016/j.peva.2010.08.009

[9] Ranganathan P, Leech P, Irwin D, Chase J. Ensemble-level Power Management for Dense Blade Servers. Proc of the ACM International Symposium on Computer Architecture (ISCA); Boston, USA. 2006. p. 66–77.

[10] Barroso LA, Hölzle U. The Case for Energy-Proportional Computing. IEEE Computer. 2007; 40: 33–37. doi: 10.1109/MC.2007.443

[11] Meisner D, Gold BT, Wenisch TF. PowerNap: Eliminating Server Idle Power. Proc of the ACM Architectural Support for Programming Languages and Operating Systems (ASPLOS); Washington, D.C., USA. 2009. p. 205–216.

[12] Meisner D, Sadler CM, Barroso LA, Weber WD, FWenisch T. Power Management of Online Data-Intensive Services. Proc of the ACM International Symposium on Computer Architecture (ISCA); San Jose, USA. 2011. p. 319–330.

[13] Li C, Zhou R, Li T. Enabling distributed generation powered sustainable high-performance data center. High Performance Computer Architecture (HPCA2013), 2013 IEEE 19th International Symposium; IEEE. 2013. p. 35–46.

[14] Li C, Zhang W, Cho CB, Li T. Solarcore: Solar energy driven multi-core architecture power management. 2011 IEEE 17th International Symposium on High Performance Computer Architecture; IEEE. 2011. p. 205–216.

[15] Aksanli B, Rosing T, Pettis E. Distributed battery control for peak power shaving in datacenters. Green Computing Conference (IGCC), 2013 International; IEEE. 2013. p. 1–8.

[16] Aksanli B, Pettis E, Rosing T. Architecting efficient peak power shaving using batteries in data centers. 2013 IEEE 21st International Symposium on Modelling, Analysis and Simulation of Computer and Telecommunication Systems; IEEE. 2013. p. 242–253.

[17] Govindan S, Sivasubramaniam A, Urgaonkar B. Benefits and Limitations of Tapping into Stored Energy for Datacenters. Proc of the ACM International Symposium on Computer Architecture (ISCA); San Jose, USA. 2011. p. 341–352.

[18] Ton M, Fortenbury B. High Performance Buildings: Data Centers Uninterruptible Power Supplies (UPS). 2005. Available from: http://hightech.lbl.gov/documents/ups/final_ups_report.pdf

[19] Hu X, Zou C, Zhang C, Li Y. Technological developments in batteries: A survey of principal roles, types, and management needs. IEEE Power and Energy Magazine. 2017; 15(5): 20–31. doi: 10.1109/MPE.2017.2708812

[20] Linden D, Reddy TB. Handbook of Batteries. McGraw-Hill; 2002.

[21] Pedram M, Chang N, Kim Y, Wang Y. Hybrid electrical energy storage systems. Low-Power Electronics and Design (ISLPED), 2010 ACM/IEEE International Symposium; IEEE. 2010. p. 363–368.

[22] Chen H, Cong TN, Yang W, Tan C, Li Y, Ding Y. Progress in Electrical Energy Storage System: A Critical Review. Progress in Natural Science. 2009; 19: 291–312. doi: 10.1016/j.pnsc.2008.07.014

[23] Zou C, Hu X, Wei Z, Tang X. Electrothermal dynamics-conscious lithium-ion battery cell-level charging management via state-monitored predictive control. Energy. 2017; 141(Supplement C): 250–259. doi: 10.1016/j.energy.2017.09.048

[24] Hu X, Martinez CM, Yang Y. Charging, power management, and battery degradation mitigation in plug-in hybrid electric vehicles: A unified cost-optimal approach. Mechanical Systems and Signal Processing. 2017; 87(Part B): 4–16. doi: 10.1016/j.ymssp.2016.03.004

[25] Zhang L, Wang Z, Hu X, Sun F, Dorrell DG. A comparative study of equivalent circuit models of ultracapacitors for electric vehicles. Journal of Power Sources. 2015; 274(Supplement C): 899–906. doi: 10.1016/j.jpowsour.2014.10.170

[26] Zhang L, Hu X, Wang Z, Sun F, Dorrell DG. Fractional-order modeling and state-of-charge estimation for ultracapacitors. Journal of Power Sources. 2016; 314(Supplement C): 28–34. doi: 10.1016/j.jpowsour.2016.01.066

[27] Fortenbery B, EPRI EC, Tschudi W. DC Power for Improved Data Center Efficiency. 2008. Available from: http://hightech.lbl.gov/documents/data_centers/dcdemofinalreport.pdf

[28] Google Inc. Google Datacenter Video Tour. 2009. Available from: http://www.google.com/about/datacenters/efficiency/external/2009-summit.html

[29] Miller R. Microsoft Reveals its Specialty Servers, Racks. 2011. Available from: http://www.datacenterknowledge.com/archives/2011/04/25/microsoft-reveals-its-specialty-servers-racks/

[30] Facebook Inc. Open Compute Project. 2011. Available from: http://www.opencompute.org/

[31] Liu L, Li C, Sun H, Hu Y, Gu J, Li T, et al. Heb: deploying and managing hybrid energy buffers for improving datacenter efficiency and economy. ACM SIGARCH Computer Architecture News vol. 43; ACM. 2015. p. 463–475.

[32] Koushanfar F. Hierarchical hybrid power supply networks. Proceedings of the 47th Design Automation Conference; ACM. 2010. p. 629–630.

[33] Ramadass P, Haran B, White R, Popov BN. Mathematical modeling of the capacity fade of li-ion cells. Journal of Power Sources. 2003; 123(2): 230–240. doi: 10.1016/S0378-7753(03)00531-7

[34] Los Angeles Department of Water & Power, Electric Rates. Available from: http://www.ladwp.com/ladwp/cms/ladwp001752.jsp

[35] Time of Use Hours & Pricing. Available from: https://www.pacificpower.net/ya/po/otou/ooh.html

[36] Consolidated Edison Company of New York, Inc., Service Classification No. 1—Residential and Religious. 2012.

[37] Kok JK, Warmer CJ, Kamphuis I. Powermatcher: multiagent control in the electricity infrastructure. Proceedings of the fourth international joint conference on Autonomous agents and multiagent systems; ACM. 2005. p. 75–82.

[38] Xie Q, Lin X, Wang Y, Pedram M, Shin D, Chang N. State of Health Aware Charge Management in Hybrid Electrical Energy Storage Systems. Proc of the Conference on Design, Automation and Test in Europe (DATE); Dresden, Germany. 2012. p. 1060–1065.

[39] Wang Y, Lin X, Chang N, Pedram M. Dynamic reconfiguration of photovoltaic energy harvesting system in hybrid electric vehicles. Proceedings of the 2012 ACM/IEEE international symposium on Low power electronics and design; ACM. 2012. p. 109–114.

[40] Cormen TH. Introduction to algorithms. MIT press; 2009.

[41] Google cluster data. 2012. Available from: https://code.google.com/p/googleclusterdata/

[42] Pedram M, Hwang I. Power and performance modeling in a virtualized server system. Green Computing Workshop in conjunction with International Conference on Parallel Processing. 2010.

